# Inverse-probability weighting and multiple imputation for evaluating selection bias in the estimation of childhood obesity prevalence using data from electronic health records

**DOI:** 10.1186/s12911-020-1020-8

**Published:** 2020-01-20

**Authors:** Carmen Sayon-Orea, Conchi Moreno-Iribas, Josu Delfrade, Manuela Sanchez-Echenique, Pilar Amiano, Eva Ardanaz, Javier Gorricho, Garbiñe Basterra, Marian Nuin, Marcela Guevara

**Affiliations:** 1 Servicio Navarro de Salud, Pamplona, Spain; 2 Department of Preventive Medicine and Public Health, University of Navarra, Pamplona, Spain; 3 Public Health Institute of Navarra, IdiSNA, Leyre 15, 31003 Pamplona, Spain; 4 Research Network for Health Services in Chronic Diseases (REDISSEC), Pamplona, Spain; 5 Biomedical Research Center Network for Epidemiology and Public Health (CIBERESP), Madrid, Spain; 6 Primary Healthcare, Navarra Health Service, Pamplona, Spain; 7 Public Health Division of Gipuzkoa, Department of Health of the Basque Government, Donostia-San Sebastian, Gipuzkoa Spain; 8 Department of Health, Navarra Regional Government, Pamplona, Spain

**Keywords:** Inverse-probability weighting, Multiple imputation, Childhood obesity, Weight status, Prevalence, Electronic health records

## Abstract

**Background and objectives:**

Height and weight data from electronic health records are increasingly being used to estimate the prevalence of childhood obesity. Here, we aim to assess the selection bias due to missing weight and height data from electronic health records in children older than five.

**Methods:**

Cohort study of 10,811 children born in Navarra (Spain) between 2002 and 2003, who were still living in this region by December 2016. We examined the differences between measured and non-measured children older than 5 years considering weight-associated variables (sex, rural or urban residence, family income and weight status at 2–5 yrs). These variables were used to calculate stabilized weights for inverse-probability weighting and to conduct multiple imputation for the missing data. We calculated complete data prevalence and adjusted prevalence considering the missing data using inverse-probability weighting and multiple imputation for ages 6 to 14 and group ages 6 to 9 and 10 to 14.

**Results:**

For 6–9 years, complete data, inverse-probability weighting and multiple imputation obesity age-adjusted prevalence were 13.18% (95% CI: 12.54–13.85), 13.22% (95% CI: 12.57–13.89) and 13.02% (95% CI: 12.38–13.66) and for 10–14 years 8.61% (95% CI: 8.06–9.18), 8.62% (95% CI: 8.06–9.20) and 8.24% (95% CI: 7.70–8.78), respectively.

**Conclusions:**

Ages at which well-child visits are scheduled and for the 6 to 9 and 10 to 14 age groups, weight status estimations are similar using complete data, multiple imputation and inverse-probability weighting. Readily available electronic health record data may be a tool to monitor the weight status in children.

## Background

According to the World Health Organization, childhood obesity is a serious public health challenge of the twenty-first century [1]. In 2010, it was estimated that 42 million children under 5 years of age were overweight worldwide. Obesity is associated with conditions such as adverse cardiovascular and metabolic outcomes, mental health and psychological issues, and/or respiratory and orthopedic problems. Furthermore, childhood obesity increases the likelihood of obesity in adulthood [2]. Public health programs are currently focused on monitoring the trend in prevalence of overweight by sex and age, as well as assessing geographic ethnic, or socioeconomic inequalities to help improve the policies and interventions. Worldwide, monitoring has been carried out through national surveys, e.g., self-reported information provided by the parents or by direct measurements of weight and height, as in the case of the National Health and Nutrition Examination Surveys in the USA [3], or the WHO European Childhood Obesity Surveillance Initiative [4]. Standardized measurements of weight and height and the establishment of nationally representative samples are some of the strengths of these actions.

Over the last years, measurements of weight and height from electronic health records (EHR) databases have been used in Canadian, [5] British, [6] North American, [7] Swedish, [8] and Spanish studies [9, 10] to estimate trends in childhood obesity prevalence. The use of EHR data for surveillance does not require bespoke data collection or patient recruitment, [11] it provides information for small areas and all age groups at any time and lower cost in comparison to surveys. However, to obtain reliable results from these data, the estimations derived from EHRs should be free of potential bias.

The information available in EHRs comes from children who attend and are measured at primary care centers during well-child or office visits for any reason. Estimations of the prevalence of weight status using incomplete EHR data could be biased if there are systematic differences in characteristics associated with weight status (e.g., income, sex, age, etc.) between measured and non-measured children. It is necessary to identify the factors leading to missingness (lack of weight and height measurements in our case) and apply strategies, such as inverse-probability weighting (IPW) or multiple imputation (MI) to obtain corrected estimations [12]. When using IPW, complete cases are weighted by the inverse of their probability of being a complete case [13]. A pseudo-population is generated in which each individual receives a weight representing the inverse of the conditional probability of being measured (conditional to the actual values of the confounding variables) [14, 15]. The validity of this method relies on a correctly specified model including all possible variables associated with missingness [14]. In the same context, MI produce unbiased estimates for the parameters of interest through a simulation-based procedure that replaces each missing value with a set of plausible values, thus creating complete data sets. The results are combined in a final estimate (average) that incorporates the variability of the data and some additional variability acknowledging any uncertainty on the missing values [16].

The purpose of this study is to examine the selection bias associated with missing weight and height data from a single EHR and compare the prevalence of obesity derived from complete data and estimators obtained using IPW and MI.

## Methods

### Study population

This study was carried out in Navarra, a region in northern Spain with 640,000 inhabitants and around 6000 live births every year. The Navarra Health Service provides free healthcare access to most of the region’s population. EHRs have been used since 2000 and include reports from primary care, hospital admissions, regional vaccinations, and laboratory test results. A single EHR is used in public primary care centers where a team formed by a pediatrician, a pediatric nurse, and occasionally a social worker perform well-child visits through the Navarra Child Health Program; the first visit is at home and from then on at the primary care centers. Vaccines are administered mostly in primary care centers until the age of six and then at school. The Child Health Program includes 15 health exams: nine during the first 3 years of life, and one at three, four, six, eight, 11, and 14 years. Weight and height, are measured during the visits using standardized methods and, in most cases, these measurements, together with the date at which they were performed, are stored as structured data in the EHRs making data mining easier. A software based on OMI-AP is used for managing the primary care EHR, [17] which is organized in a structured list (bio-psycho-social problems, reason for consultation, etc.) following the International Classification of Primary Care, Second Edition (ICPC-2).

### Assessment of selection bias

We first determined the percentage of children with healthcare cards of the Navarra Health Service who had height and weight measurements in their first 14 years of life. By December 31, 2016, there were 95,321 children under 15 years of age in the primary EHR database from a total of 100,301 inhabitants of that age. Weight and height measurements data stored in EHR by the pediatric team during the visits were used to estimate the percentages by age of children with at least one measurement within one-year period (from January to December of 2016) and four-year period (2013–2016) (Table 1). The lowest percentages of measurements in one-year periods were seen for ages when well-child visits were not scheduled (5, 7, 9, 10, 12, and 13 years). When considering longer time-frames (4 years), the percentages of measured children were around 97–99% for children under 5 years. Thus, weight status estimates obtained using the data of these children are generalizable to the whole population of the same age. For valid overweight and obesity prevalence estimations in children older than 5 years we had to make sure that relevant characteristics related to weight status categories were not different for measured and not measured children. In case they were different, we had to correct the possible selection bias. Since there was no other sources to validate EHR height and weight data (e.g., a Nutrition Examination Survey), we analyzed if the information of the measured children older than 5 years was biased in relation to a group of variables, specifically sex, family income, urban/rural residence, and weight status at ages 2–5 years, for whom this information was available for almost all children included in the study.

**Table 1:** Number and percentage of children with weight and length/height data in the electronic health record by age and one- and four-year periods

Age by December 2016 (years)		Weight and length/height within a one-year period (2016)		Weight and length/height within a four-year period (2013–2016)	
	N	n	%	n	%
0–12 months^a^	5570	5387	96.7%	5387	96.7%
1 year^a^	5953	5780	97.1%	5839	98.1%
2 years^a^	5940	5624	94.7%	5898	99.3%
3 years^a^	5874	4996	85.1%	5836	99.4%
4 years^a^	6411	4992	77.9%	6307	98.4%
5 years	6541	1231	18.8%	6342	97.0%
6 years^a^	6564	5119	78.0%	6332	96.5%
7 years	6593	1323	20.1%	6239	94.6%
8 years^a^	6822	4999	73.3%	6415	94.0%
9 years	6605	1728	26.2%	6125	92.7%
10 years	6717	1923	28.6%	5979	89.0%
11 years^a^	6397	3719	58.1%	5808	90.8%
12 years	6518	1978	30.4%	5684	87.2%
13 years	6588	1147	17.4%	5629	85.4%
14 years^a^	6228	3628	58.3%	5243	84.2%
Total	95,321	53,574	56.2%	89,063	93.4%

^a^The Child Health Program recommends weight and height measurements at this age

#### Outcomge variable

When IPW was used, measured/non-measured was the outcome variable. Having height and weight data for the same time point for ages 6 to 14 years and for 6–9 and 10–14 age groups was considered as being measured. Missing data for height and/or weight for the same time point was considered as being not-measured.

#### Explanatory variables

Sex (boy/girl), rural/urban residence (urban residence included cities with ≥35,000 inhabitants; rural residence included towns with < 35,000 inhabitants), annual family income (ordinal categories from 1 to 4 [1: basic income (families living on minimum subsistence income), 2: < 18,000 €, 3: 18,000 to < 100,000 €, and 4: ≥ 100,000 €), [18] and weight status at 2–5 years (underweight, normal weight, at risk of overweight and overweight/obese). Latest body mass index (BMI) at 2–5 years was calculated as the weight in kilograms divided by height in meters squared (kg/m^2^). BMI z-scores were determined using the WHO standards [19–21]. We categorized the weight status as follows: underweight (< − 2 SD), normal weight (≥ − 2 to ≤1 SD), at risk of overweight (> 1 to ≤2 SD), overweight/obese (> 2 SD). For older children, the cut-off for underweight was < − 2 SD), normal weight ≥ − 2 to ≤1 SD, overweight + 1 SD and obesity + 2 SD [22].

**Fig. 1:**
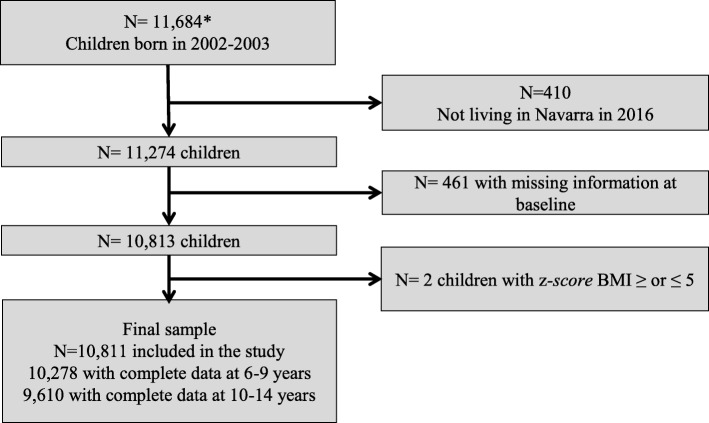
Flowchart. * Children born between 2002 and 2003 in Navarra and included in the EHR of primary care

Children for whom there were no measurements at ages 2–5 years and those with missing values of variables used as potential predictors of missing data or implausible z-scores of BMI values (≤ 5 SD and ≥ 5 SD) were excluded from the study. Thus, 10,811 children born between 2002 and 2003 that were living in Navarra by December 2016 (aged 13–14 years at that time) were included (Fig. 1).

### Statistical analysis

We used IPW to determine whether missing data introduce bias when estimating weight status prevalence from EHRs. We fit a logistic regression model to find which variables were associated with “no weight and height measurements” for each age (between 6 and 14 years) and for two age intervals: 6–9 and 10–14 years. We included the following variables in the model: sex, weight status at 2–5 years, rural/urban residence, and annual household income.

To adjust for selection bias, a set of stabilized weights were computerized, with the probability of having complete data (measured at all ages and in the two age groups: 6–9 and 10–14 years) in the numerator and other variables associated with missing or complete data. The numerator was calculated directly from the data as the probability of being measured at the analyzed age. The denominator was computerized using logistic regression with complete data (yes/no) as the outcome and factors associated to missing data as independent variables. Robust variance estimate was used to calculate IPW. Mean standard deviation of weights was 0.09. Weighted proportions were calculated using the [*pweight*] statement.

$$ SW=\frac{f\left( measured\ast \right)}{f\left( measured\ast | sex, weight\kern0.17em status\; at\;2-5 yr, household\kern0.17em income, rural/ urban\kern0.17em residence\right)} $$

*for each year from 6 to 14 years or for the 6–9 and 10–14 periods.

In addition to the IPW, we also used multiple imputation with the 6–9 and 10–14 age groups. An iterative Markov chain Monte Carlo method (STATA “mi” command) was applied. We generated 10 imputations for each missing measurement. The imputation models included sex, weight status at 2–5 years, rural/urban residence, and annual household income as predictors.

We calculated the prevalence for complete data, and IPW and MI estimated prevalence for each weight status at different ages and age groups.

All analyses were conducted with STATA, version 12.0 (Stata Corp).

## Results

Ninety-five percent and 88.9% of measured 2-to-5-year old children had one or more measurements at 6–9 and 10–14 years, respectively. Measurement status based on the characteristics of the children for age groups 6–9 and 10–14 years are shown in Tables 2 and 3, as well as the results of the logistic regression analysis. At 6–9 years, being a girl (OR = 1.18 (95% CI: 0.99–1.42), being at risk of overweight (OR = 1.29 (95% CI: 1.02–1.63), and having an annual household income of 18,000 and < 100,000 € (OR = 1.70 (95% CI: 1.41–2.06) were associated with higher probability of being measured; while at ages 10–14 years the variables were being a girl (OR = 1.15 (95% CI:1.02–1.29) and having an annual household income of 18,000 to < 100,000 € (OR = 1.38 (95% CI: 1.22–1.57). On the other hand, belonging to a family with basic income (OR = 0.40 (95% CI: 0.31–0.54) and living in an urban area (OR = 0.79 (95% CI: 0.66–0.94) were associated with lower probability of being measured at the age of 6–9 years; being underweight (OR = 0.53 (95% CI:0.30–0.92) and belonging to a family with basic household income (OR = 0.57 (95% CI: 0.45–0.72) decreased the probability of being measured at the age of 10–14 years. Multivariate logistic regression model results for every age are shown in Additional file 1: Tables S1-S9. In ages where well-child visits were scheduled, the variable that was consistently and positively associated with having height and weight measurements was an annual household income from 18,000 to < 100,000 €. At ages at which well-child check-ups were not scheduled (7, 9, 10, 12, and 13) being overweight/obese at 2–5 years was positively associated with the existence of measurements in the EHR.

**Table 2:** Odds ratio and 95% confidence interval (OR 95% CI) of having height and weight measurements at ages 6–9 years based on the characteristics of the children

	Total *n* = 10,811	Measured *n* = 10,278 (95.1)	Not measured *n* = 533 (4.9)	Adjusted OR^a^
Sex				
Boys	5498	5203 (94.6)	295 (5.4)	1 (ref.)
Girls	5313	5075 (95.5)	238 (4.5)	1.18 (0.99–1.42)
Weight status at 2–5 years of age				
Underweight	78	70 (89.7)	8 (10.3)	0.52 (0.25–1.11)
Normal weight	7628	7241 (94.9)	387 (5.1)	1 (ref.)
At risk of overweight	2291	2198 (95.9)	93 (4.1)	1.29 (1.02–1.63)
Overweight/ Obese	814	769 (84.5)	45 (5.5)	0.99 (0.72–1.37)
Annual household income				
Basic income	510	442 (86.7)	68 (13.3)	0.40 (0.31–0.54)
< 18,000 €	4657	4390 (94.3)	267 (5.7)	1 (ref.)
18.000 € - < 100.000 €	5546	5352 (96.5)	194 (3,5)	1.70 (1.41–2.06)
> 100,000 €	97	93 (95.9)	4 (4.1)	1.45 (0.53–3.98)
Residence				
Rural	6447	6159 (95.5)	288 (4.5)	1 (ref.)
Urban	4364	4119 (94.4)	245 (5.6)	0.79 (0.66–0.94)

Values of measured and not measured are expressed as numbers (%)

^a^ Adjusted simultaneously for all variables in the table. *OR* odds ratio

**Table 3:** Odds ratio and 95% confidence interval (OR 95%CI) of having height and weight measurements at ages 10–14 years based on the characteristics of the children

	Total *n* = 10,811	Measured *n* = 9610 (88.9)	Not measured *n* = 1201 (11.1)	Adjusted OR^a^
Sex				
Boys	5498	4849 (88.2)	649 (11.1)	1 (ref.)
Girls	5313	4761 (89.6)	552 (10.4)	1.15 (1.02–1.29)
Weight status at 2–5 years of age				
Underweight	78	62 (79.5)	16 (20.5)	0.53 (0.30–0.92)
Normal weight	7628	6763 (88.7)	865 (11.3)	1 (ref.)
At risk of overweight	2291	2057 (89.8)	234 (10.2)	1.14 (0.97–1.32)
Overweight/Obese	814	728 (89.4)	86 (10.6)	1.13 (0.90–1.44)
Annual household income				
Basic income	510	409 (80.2)	101 (19.8)	0.57 (0.45–0.72)
< 18,000€	4657	4085 (87.8)	572 (12.3)	1 (ref.)
18.000€ - < 100.000 €	5546	5035 (90.8)	511 (9.2)	1.38 (1.22–1.57)
> 100,000 €	97	80 (82.5)	17 (17.5)	0.66 (0.39–1.12)
Residence				
Rural	6447	5735 (89.0)	712 (11.0)	1 (ref.)
Urban	4364	3875 (88.8)	489 (11.2)	0.99 (0.87–1.12)

Values of measured and not measured are expressed as numbers (%)

^a^ Adjusted simultaneously for all variables in the table. *OR* odds ratio

**Fig. 2:**
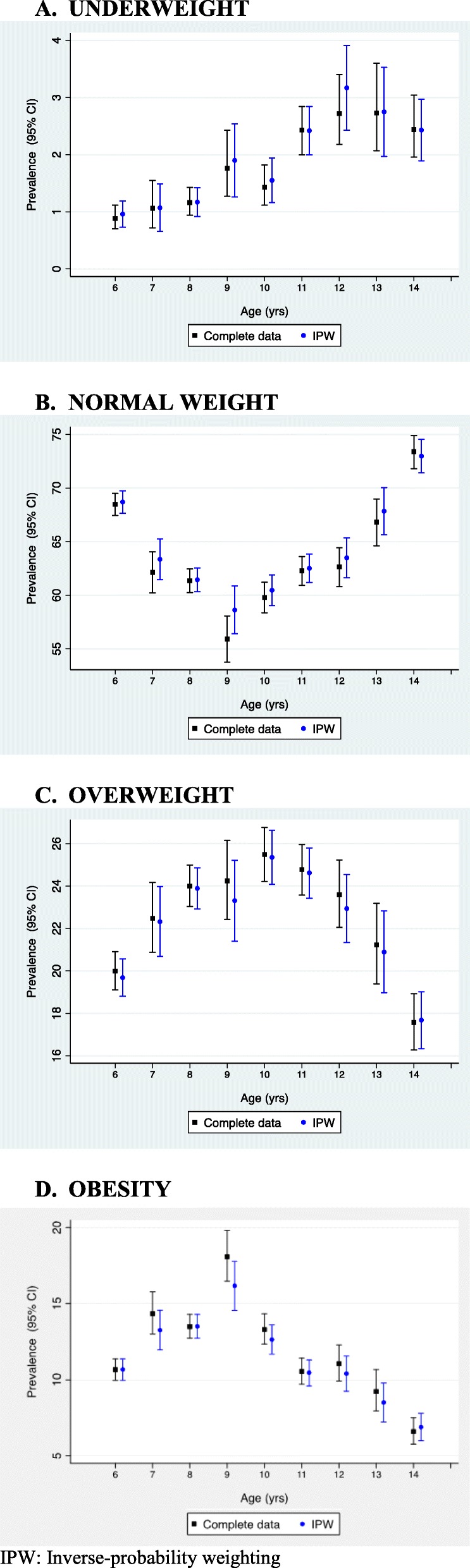
Weight status for ages 6 to 14 estimated for complete data in EHRs and adjusted for missing data using IPW

**Fig. 3:**
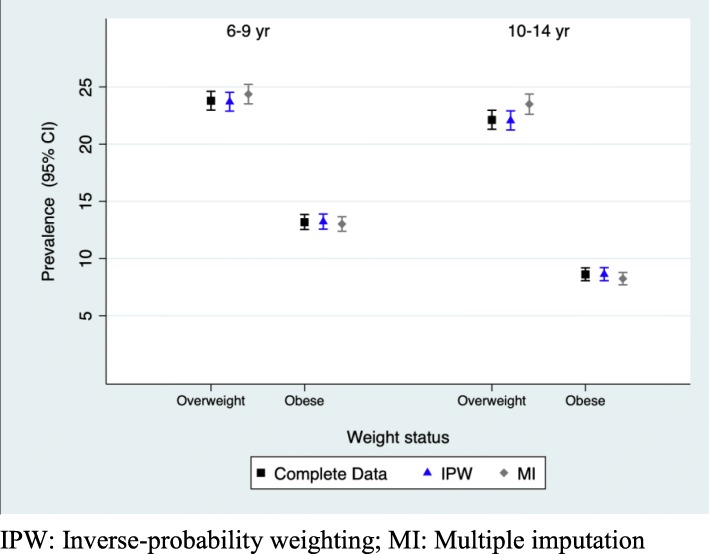
Weight status for 6–9 and 10–14 years age groups estimated for complete data in EHRs and adjusted for missing data using IPW and MI. IPW: Inverse-probability weighting; MI: Multiple imputation

Figure 2 and Additional file 1: Table S10 show the prevalence of underweight, normal weight, overweight and obesity estimated for complete data in EHRs and adjusted for missing data using IPW for ages 6 to 14. Table 4 and Fig. 3 show the prevalence of weight status for age groups 6–9 and 10–14 estimated for complete data and adjusted for missing data using MI and IPW. As shown in Fig. 2 the 95% CI between complete data and IPW prevalence completely overlap in all the estimations. At ages at which well-child visits were not scheduled in the Childhood Health Program (7, 9, 10, 12 and 13 years), the prevalence for complete data in EHRs overestimates the prevalence of obesity, although the differences were not statistically significant. When prevalence were calculated for longer age intervals (6–9 and 10–14 years), complete data, IPW and MI weight status estimators did not differ significantly. At 6–9 years complete data, IPW and MI obesity age-adjusted prevalence were 13.18% (95% CI: 12.54–13.85), 13.22% (95% CI: 12.57–13.89) and 13.02% (95% CI: 12.38–13.66) and at 10–14 years 8.61% (95% CI: 8.06–9.18), 8.62% (95% CI: 8.06–9.20) and 8.24% (95% CI:7.70–8.78), respectively.

**Table 4:** Weight status for 6–9 and 10–14 years age groups estimated for complete data in EHRs and adjusted for missing data using IPW and MI

	Underweight	Normal weight	Overweight	Obese
6–9 years; *n* = 10,278				
Complete data	1.22% (1.02–1.45)	61.81% (60.87–62.75)	23.79% (22.98–24.62)	13.18% (12.54–13.85)
IPW	1.24% (1.04–1.47)	61.84% (60.90–62.78)	23.70% (22.89–24.53)	13.22% (12.57–13.89)
MI	1.53% (1.27–1.79	61.08% (60.12–62.03)	24.37% (23.52–25.22)	13.02%(12.38–13.66)
10–14 years; *n* = 9610				
Complete data	2.16% (1.89–2.47)	67.11% (66.16–68.04)	22.12% (21.30–22.96)	8.61% (8.06–9.18)
IPW	2.18% (1.91–2.50)	67.14% (66.19–68.07)	22.06% (21.24–22.91)	8.62% (8.06–9.20)
MI	2.90% (2.49–3.30)	65.36% (64.42–66.30)	23.50% (22.61–24.38)	8.24% (7.70–8.78)

*IPW* Inverse-probability weighting; *MI* Multiple imputation

## Discussion

Our results show that weight and height data stored in primary care EHRs are highly valuable for childhood obesity surveillance in our population despite measurement under-representation for certain subpopulations. Non-measured children are more likely to belong to a family with basic income and being underweight at early age. On the other hand, being a girl, being at risk overweight in early age and having an annual household income ≥18,000 € increases the probability of being measured. Finally, missingness does not introduce significant selection bias for estimating weight status prevalence when the data is grouped by age range (6–9 and 10–14) or at ages 6, 8, and 14 years at which the Childhood Health Program recommended measuring height and weight.

Thorough measurements of children’s weight and height decreases with age, particularly ages at which there are no scheduled visits as established by the Navarra Childhood Health Program. The higher number of visits scheduled during the first years of the child’s life and the fact that vaccines are administered in primary care centers until the age of six may explain the larger percentage of children under 3 years for whom weight and height measurements are available in comparison with older ages. The decrease in the number of health exams scheduled after the age of three and the fact that vaccines are administered mostly at schools influences the observed decline at higher ages. Studies in which the prevalence of childhood obesity using EHRs have been assessed conclude that the use of this tool for obesity surveillance in children might be feasible but suggest that further studies are needed to determine the validity and the reliability of EHR data, [5] particularly because at older ages there is a dramatic decrease of available measurements. In our population, a major concern was determining the representativeness of the weight status data extracted from the EHRs in the case of older children. We hypothesized that at older ages different characteristics such as sex, socioeconomic status, place of residence, and even the weight status could affect the probability of being measured. Consequently, weight status prevalence estimations obtained from complete data cannot necessarily be generalized to the population. We observed that children from poorer families and being boys, both factors associated with higher prevalence of obesity, were underrepresented in age ranges 6–9 and 10–14 years, which can result in an underestimation of obesity prevalence. On the other hand, two to five-year children being at risk of overweight or living in rural areas, which also have higher prevalence of obesity, are overrepresented in the EHRs. The fact that different selection bias are acting in opposite directions explains result similarities in complete data, IPW and MI prevalence estimators. It should also be noted that the highest differences between complete data and IPW overweight and obesity prevalence are seen at ages at which there were no scheduled Childhood Health Program visits (i.e., seven, nine, 10, 12, and 13 years). Furthermore, children at these ages with overweight/obesity are overrepresented probably because the pediatric team carries out additional measurements. When greater age ranges are used for weight status estimations, a large percentage of children are included in the calculations, and complete data and IPW and MI estimators that account for bias due to missing data were similar. For overweight and obesity prevalence estimations by age group (i.e., 6–9 and 10–14 years), measurement percentages (95.1 and 88.9%, respectively) are greater than those achieved by the National Study of Prevalence of Overweight and Obesity in Spanish Children in 2011 (75.5%) [23]. Within this context, a Swedish study aiming to find the variables associated to the low representation of children in a health survey observed that those of single parent families, foreign background, and poorer education and income were underrepresented in the study [24]. All variables were associated with a risk of overweight and obesity, therefore, an adjustment of complete data estimations was necessary.

There is no increase in the probability of being measured at the age range 6–9 years of children with overweight or obesity at early years unlike those being at risk of overweight at 2–5 years. This can be partly explained by the fact that these children could be treated in specialized care, specifically in pediatric endocrinology. It can also indicate that parents of children with obesity miss well-care visits to avoid receiving advice on their children’s weight, as some studies have pointed out [25]. It has been shown that many healthcare providers hold strong negative attitudes and stereotyped beliefs toward people with obesity. Furthermore, considerable evidence suggests that such attitudes influence the perception of the individual and may lead to avoidance of care [26]. Other characteristics, such as children of emigrants -not considered in this study- and are related with the prevalence of obesity, may help explain this finding.

In a work that is similar to the one presented here, Funk et al. [27] conducted a study to assess the feasibility of using BMI from EHRs to estimate overweight and obesity prevalence and compare it to data from the National Health Survey and used IPW to adjust the prevalence. The authors found resemblances in the estimation of crude and adjusted prevalence to those of the National Health Survey, concluding that EHRs might be the ideal tool to identify and target patients with obesity and implement public health interventions. Moreover, a study conducted in the UK provides proof-of-concept for the use of primary care EHRs to assess children’s obesity in England, suggesting it is a valuable resource for monitoring obesity trends [28].

A cross-sectional survey conducted in the Basque Country in 2015, a region bordering with Navarra, directly measured weight and height and found that overweight and obesity prevalence in children between 6 and 9 years seems to be quite similar (22.90 (95% CI: 19.8–26.0) and 11.27 (95% CI: 8.8–13.5), respectively) to those obtained in Navarra from the EHRs (23.70 (95% CI: 22.89–24.53) and (13.22 (95% CI: 12.57–13.89)) [29]. These data are in line with the pattern observed in the 2006–2007 National Health Survey for these two regions [30].

Unlike in other countries, primary care EHRs in Navarra include well-child visit measurements that contribute significantly to reduce biases and increase the reliability of weight status prevalence estimations. Another strength is the widespread use of EHRs by pediatric teams. Finally, the fact that most of the infant population is included in this single EHR ensures the generalizability to the total population.

There are some potential limitations regarding EHR data. On the one hand, the introduced information involves a number of different healthcare professionals, and therefore, there may be some variability regarding the measurement method. Another limitation is that the professionals manually enter weight and height values and this is less reliable than the data entered for research, where there is double data entry and is subjected to validity checks. To avoid implausible values, z-scores values ≤5 SD and ≥ 5 SD were excluded. A strength of EHR data is the large number of children included; this allows obtaining estimations for large and small areas, as well as for all age groups. In Spain, few national surveys provide estimations for children between 6 and 9 years [23, 31, 32]; these national studies allow data comparisons against other European studies, but not estimations for certain Spanish regions due to the low number of subjects.

Some authors believe that the best place for measuring height and weight is in primary care settings during well-child visits, ensuring the appropriate equipment is used, continuous training of the staff, and the use of measurement protocols [33]. Furthermore, these settings minimize unintended negative consequences associated to monitoring growth in other environments (e.g., schools), such as stigmatization.

Another limitation of this study is that certain variables potentially associated to a lack of measurements, such as ethnicity or mono-parental status, among others, were not available.

## Conclusions

Missing height and weight data in the EHR did not introduce significant selection bias in weight status estimations for the 6–9 and 10–14 year age groups. Readily available EHR data could be a valid tool for surveillance of weight status in our population. Therefore, in our population, EHRs are a potentially cost-effective, emerging tool for public health surveillance.

## Additional file

**Additional file 1: Table S1**. Odds Ratio and 95% confidence interval (OR 95%CI) of complete data at 6 years according to baseline characteristics. **Table S2**. Odds Ratio and 95% confidence interval (OR 95%CI) of complete data at 7 years according to baseline characteristics. **Table S3.** Odds Ratio and 95% confidence interval (OR 95%CI) of complete data at 8 years according to baseline characteristics. **Table S4.** Odds Ratio and 95% confidence interval (OR 95%CI) of complete data at 9 years according to baseline characteristics. **Table S5.** Odds Ratio and 95% confidence interval (OR 95%CI) of complete data at 10 years according to baseline characteristics. **Table S6.** Odds Ratio and 95% confidence interval (OR 95%CI) of complete data at 11 years according to baseline characteristics. **Table S7.** Odds Ratio and 95% confidence interval (OR 95%CI) of complete data at 12 years according to baseline characteristics. **Table S8.** Odds Ratio and 95% confidence interval (OR 95%CI) of complete data at 13 years according to baseline characteristics. **Table S9.** Odds Ratio and 95% confidence interval (OR 95%CI) of complete data at 14 years according to baseline characteristics. **Table S10.** Complete data and IPW adjusted prevalence and 95% confidence interval for every age between 6 to 14 years.

## Data Availability

The datasets used and/or analyzed during this study are available from the corresponding author upon reasonable request.

## Abbreviations

BMI: Body mass index
CI: Confidence intervals
EHRs: Electronic health records
ICPC-2: International Classification of Primary Care, 2nd Edition
IPW: Inverse-probability weighting
MI: Multiple Imputation
SD: Standard deviation
WHO: World Health Organization
