# Supplementary material for: Inverse-probability weighting and multiple imputation for evaluating selection bias in the estimation of childhood obesity prevalence using data from electronic health records
Source: BMC Med Inform Decis Mak. 2020 Jan 20;20:9. doi: 10.1186/s12911-020-1020-8 (PMC6971988; doi:10.1186/s12911-020-1020-8)
Supplement: Supplementary file 1 — Additional file 1: Table S1. Odds Ratio and 95% confidence interval (OR 95%CI) of complete data at 6 years according to baseline characteristics. Table S2. Odds Ratio and 95% confidence interval (OR 95%CI) of complete data at 7 years according to baseline characteristics. Table S3. Odds Ratio and 95% confidence interval (OR 95%CI) of complete data at 8 years according to baseline characteristics. Table S4. Odds Ratio and 95% confidence interval (OR 95%CI) of complete data at 9 years according to baseline characteristics. Table S5. Odds Ratio and 95% confidence interval (OR 95%CI) of complete data at 10 years according to baseline characteristics. Table S6. Odds Ratio and 95% confidence interval (OR 95%CI) of complete data at 11 years according to baseline characteristics. Table S7. Odds Ratio and 95% confidence interval (OR 95%CI) of complete data at 12 years according to baseline characteristics. Table S8. Odds Ratio and 95% confidence interval (OR 95%CI) of complete data at 13 years according to baseline characteristics. Table S9. Odds Ratio and 95% confidence interval (OR 95%CI) of complete data at 14 years according to baseline characteristics. Table S10. Complete data and IPW adjusted prevalence and 95% confidence interval for every age between 6 to 14 years. [file 12911_2020_1020_MOESM1_ESM.docx]

**SUPPLEMENTARY MATERIAL.**

*STATA code used to create the stabilized weights for IPW*

Inverse probability weights were calculated at each age from 6 until 14 years, and also for 6-9 year period and 10-14 period, using the following statements in STATA:

logit *vardep* *var1 var2 var3 var4*, or

predict p_cond ##conditioned probability

replace p_cond=1-p_cond6 if *vardep*==0

sum *vardep*

gen P_marg=r(mean) ## marginal probability

replace P_marg=1-P_marg if *vardep*==0

g IPW=P_marg/p_cond

proportion weight_status

proportion weight_status [pweight = IPW6]

**Supplementary Table 1.  Odds Ratio  and 95% confidence interval ( OR 95%CI) of complete data at 6 years    according to baseline characteristics**

|  | **Total**  **n = 10,811**  **n (%)** | **Measured**  **n = 7,688 (71.1)**  **n (%)** | **Not measured**  **n = 3123**  **(28.9)**  **n (%)** | **Adjusted OR^a^** |
| --- | --- | --- | --- | --- |
| **Sex** |  |  |  |  |
| **Boys** | 5,498 | 3,888 (70.7) | 1,610 (29.3) | 1 (ref.) |
| **Girls** | 5,313 | 3,800 (71.5) | 1,513 (28.5) | 1.03 (0.95 - 1.12) |
| **Weight status at 2-5 years of age** |  |  |  |  |
| **Underweight** | 78 | 42 (53.8) | 36 (46.2) | 0.50 (0.32 – 0.79) |
| **Normal weight** | 7,628 | 5,386 (70.6) | 2242 (29.4) | 1 (ref.) |
| **At risk of overweight** | 2,291 | 1,692 (73.8) | 599 (26.2) | 1.19 (1.07 - 1.32) |
| **Overweight/Obese** | 814 | 568 (69.8) | 246 (30.2) | 0.99 (0.85 - 1.16) |
| **Annual household income** |  |  |  |  |
| **Basic income** | 510 | 291 (57.1) | 219 (42.9) | 0.60 (0.49 - 0.72) |
| **Less than 18,000 €** | 4,657 | 3,234 (69.4) | 1,423 (30.6) | 1 (ref.) |
| **18.000€ - < 100.000 €** | 5,546 | 4,089 (73.7) | 1457 (26.3) | 1.24 (1.14 – 1.35) |
| **> 100,000 €** | 97 | 74 (76.3) | 23 (23.7) | 1.44 (0.89 – 2.31) |
| **Area of residence** |  |  |  |  |
| **Rural** | 6,447 | 4,253 (72.4) | 2,618 (27.6) | 1 (ref.) |
| **Urban** | 4,364 | 3,435 (69.5) | 1,505 (30.5) | 0.86 (0.79-0.93) |

^a Adjusted simultaneously for all the variables included in the table^

^OR, Odds Ratio^

**Supplementary Table 2. Odds Ratio  and 95% confidence interval ( OR 95%CI) of complete data at 7 years according to baseline characteristics**

|  | **Total**  **n = 10,811**  **n (%)** | **Measured**  **n = 2,456**  **(22.7)**  **n (%)** | **Not measured**  **n = 8,355**  **(77.3)**  **n (%)** | **Adjusted OR^a^** |
| --- | --- | --- | --- | --- |
| **Sex** |  |  |  |  |
| **Boys** | 5,498 | 1,203 (21.9) | 4,295 (78.1) | 1 (ref.) |
| **Girls** | 5,313 | 1,253 (23.6) | 4,060 (76.4) | 1.11 (1.01 - 1.21) |
| **Weight status at 2-5 years of age** |  |  |  |  |
| **Underweight** | 78 | 19 (24.4) | 59 (75.6) | 1.13 (0.67 – 1.91) |
| **Normal weight** | 7,628 | 1,693 (22.2) | 5,935 (77.8) | 1 (ref.) |
| **At risk of overweight** | 2,291 | 523 (22.8) | 1,768 (77.2) | 1.03 (0.92 - 1.15) |
| **Overweight/Obese** | 814 | 221 (27.2) | 593 (72.8) | 1.31 (1.11 – 1.55) |
| **Annual household income** |  |  |  |  |
| **Basic income** | 510 | 114 (22.4) | 396 (77.6) | 0.95 (0.76 – 1.18) |
| **Less than 18,000 €** | 4,657 | 1,069 (22.9) | 3,588 (77.1) | 1 (ref.) |
| **18.000€ - < 100.000 €** | 5,546 | 1,246 (22.5) | 4,300(77.5) | 0.97 (0.88 – 1.06) |
| **> 100,000 €** | 97 | 26 (26.8) | 71 (73.2) | 1.21 (0.77 – 1.92) |
| **Area of residence** |  |  |  |  |
| **Rural** | 6,447 | 1,264 (21.5) | 4,607 (78.5) | 1 (ref.) |
| **Urban** | 4,364 | 1,192 (24.1) | 3,748 (75.9) | 1.16 (1.06-1.27) |

^a Adjusted simultaneously for all the variables included in the table^

^OR, Odds Ratio^

**Supplementary Table 3. Odds Ratio  and 95% confidence interval ( OR 95%CI) of complete data at 8 years according to baseline characteristics**

|  | **Total**  **n = 10,811**  **n (%)** | **Measured**  **n = 7,043**  **(68.5)**  **n (%)** | **Not measured**  **n = 3,408**  **(31.5)**  **n (%)** | **Adjusted OR^a^** |
| --- | --- | --- | --- | --- |
| **Sex** |  |  |  |  |
| **Boys** | 5,498 | 3,719 (67.6) | 1,779 (32.4) | 1 (ref.) |
| **Girls** | 5,313 | 3,684 (69.3) | 1,629 (30.7) | 1.08 (0.99 - 1.17) |
| **Weight status at 2-5 years of age** |  |  |  |  |
| **Underweight** | 78 | 53 (68.0) | 25 (32.0) | 1.04 (0.64 – 1.69) |
| **Normal weight** | 7,628 | 5,205 (68.2) | 2,423 (31.8) | 1 (ref.) |
| **At risk of overweight** | 2,291 | 1,575 (68.8) | 716 (31.1) | 1.03 (0.93 - 1.14) |
| **Overweight/Obese** | 814 | 570 (70.0) | 244 (30.0) | 1.13 (0.96– 1.32) |
| **Annual household income** |  |  |  |  |
| **Basic income** | 510 | 263 (51.6) | 247 (48.4) | 0.55 (0.46 - 0.67) |
| **Less than 18,000 €** | 4,657 | 3,079 (66.1) | 1,578 (33.9) | 1 (ref.) |
| **18.000€ - < 100.000 €** | 5,546 | 3,997 (77.1) | 1,549 (27.9) | 1.33 (1.22 – 1.44) |
| **> 100,000 €** | 97 | 64 (66.0) | 33 (34.0) | 1.00 (0.65 – 1.53) |
| **Area of residence** |  |  |  |  |
| **Rural** | 6,447 | 4,072 (69.4) | 1,799 (30.6) | 1 (ref.) |
| **Urban** | 4,364 | 3,331 (67.4) | 1,609 (32.6) | 0.91 (0.83-0.98) |

^a Adjusted simultaneously for all the variables included in the table^

^OR, Odds Ratio^

**Supplementary Table 4. Odds Ratio  and 95% confidence interval ( OR 95%CI) of complete data at 9 years according to baseline characteristics**

|  | **Total**  **n = 10,811**  **n (%)** | **Measured**  **n = 2,046**  **(18.9)**  **n (%)** | **Not measured**  **n = 8,765**  **(81.1)**  **n (%)** | **Adjusted OR^a^** |
| --- | --- | --- | --- | --- |
| **Sex** |  |  |  |  |
| **Boys** | 5,498 | 983 (17.9) | 4,515 (82.1) | 1 (ref.) |
| **Girls** | 5,313 | 1,063 (20.0) | 4,250 (80.0) | 1.16 (1.05 - 1.28) |
| **Weight status at 2-5 years of age** |  |  |  |  |
| **Underweight** | 78 | 14 (18.0) | 64 (82.0) | 1.01 (0.56 – 1.82) |
| **Normal weight** | 7,628 | 1,372 (18.0) | 6,256 (82.0) | 1 (ref.) |
| **At risk of overweight** | 2,291 | 445 (19.4) | 1.846 (80.6) | 1.11 (0.98 - 1.25) |
| **Overweight/Obese** | 814 | 215 (26.4) | 599 (73.6) | 1.64 (1.39– 1.95) |
| **Annual household income** |  |  |  |  |
| **Basic income** | 510 | 93 (18.2) | 417 (81.8) | 0.91 (0.71 – 1.15) |
| **Less than 18,000 €** | 4,657 | 961 (20.6) | 3,696 (79.4) | 1 (ref.) |
| **18.000€ - < 100.000 €** | 5,546 | 975 (17.6) | 4,571 (82.4) | 0.85 (0.77 – 0.94) |
| **> 100,000 €** | 97 | 17 (17.5) | 80 (82.5) | 0.87 (0.50 – 1.47) |
| **Area of residence** |  |  |  |  |
| **Rural** | 6,447 | 1,337 (22.8) | 4,534 (77.2) | 1 (ref.) |
| **Urban** | 4,364 | 709 (14.4) | 4,231 (85.6) | 0.57 (0.51-0.63) |

^a Adjusted simultaneously for all the variables included in the table^

^OR, Odds Ratio^

**Supplementary Table 5. Odds Ratio  and 95% confidence interval ( OR 95%CI) of complete data at 10 years    according to baseline characteristics**

|  | **Total**  **n = 10,811**  **n (%)** | **Measured**  **n = 4,476 (41.4)**  **n (%)** | **Not measured**  **n = 6,335 (58.6)**  **n (%)** | **Adjusted OR^a^** |
| --- | --- | --- | --- | --- |
| **Sex** |  |  |  |  |
| **Boys** | 5,498 | 2,202 (40.1) | 3,296 (59.9) | 1 (ref.) |
| **Girls** | 5,313 | 2,274 (42.8) | 3,039 (57.2) | 1.12 (1.04 - 1.21) |
| **Weight status at 2-5 years of age** |  |  |  |  |
| **Underweight** | 78 | 15 (19.2) | 63 (80.8) | 0.35 (0.20 – 0.62) |
| **Normal weight** | 7,628 | 3,120 (40.9) | 4,508 (59.1) | 1 (ref.) |
| **At risk of overweight** | 2,291 | 941 (41.1) | 1,350 (58.9) | 1.01 (0.91 - 1.11) |
| **Overweight/Obese** | 814 | 400 (49.1) | 414 (50.9) | 1.42 (1.22– 1.63) |
| **Annual household income** |  |  |  |  |
| **Basic income** | 510 | 188 (36.9) | 322 (63.1) | 0.84 (0.70 – 1.02) |
| **Less than 18,000 €** | 4,657 | 1,909 (41.0) | 2,748 (59.0) | 1 (ref.) |
| **18.000€ - < 100.000 €** | 5,546 | 2,350 (42.4) | 3,196 (57.6) | 1.06 (0.99 – 1.15) |
| **> 100,000 €** | 97 | 29 (29.9) | 68 (70.1) | 0.62 (0.40 – 0.97) |
| **Area of residence** |  |  |  |  |
| **Rural** | 6,447 | 2,483 (42.3) | 3,388 (57.7) | 1 (ref.) |
| **Urban** | 4,364 | 1,993 (40.3) | 2,947 (59.7) | 0.92 (0.85-1.00) |

^a^ Adjusted simultaneously for all the variables included in the table

OR, Odds Ratio

**Supplementary Table 6. Odds Ratio  and 95% confidence interval ( OR 95%CI) of complete data at 11 years  according to baseline characteristics**

|  | **Total**  **n = 10,811**  **n (%)** | **Measured**  **n = 5,070 (46.9)**  **n (%)** | **Not measured**  **n = 5,741 (53.1)**  **n (%)** | **Adjusted OR^a^** |
| --- | --- | --- | --- | --- |
| **Sex** |  |  |  |  |
| **Boys** | 5,498 | 2,534 (46.1) | 2,964 (53.9) | 1 (ref.) |
| **Girls** | 5,313 | 2,536 (47.7) | 2,777 (52.2) | 1.07 (0.99 - 1.15) |
| **Weight status at 2-5 years of age** |  |  |  |  |
| **Underweight** | 78 | 43 (55.1) | 35 (44.9) | 1.47 (0.94 – 2.30) |
| **Normal weight** | 7,628 | 3,525 (46.2) | 4,103 (53.8) | 1 (ref.) |
| **At risk of overweight** | 2,291 | 1,110 (48.5) | 1,181 (51.5) | 1.10 (1.00 - 1.20) |
| **Overweight/Obese** | 814 | 392 (48.2) | 422 (51.8) | 1.10 (0.95– 1.27) |
| **Annual household income** |  |  |  |  |
| **Basic income** | 510 | 207 (40.6) | 303 (59.4) | 0.82 (0.68 – 0.99) |
| **Less than 18,000 €** | 4,657 | 2,120 (45.5) | 2,537 (54.5) | 1 (ref.) |
| **18.000€ - < 100.000 €** | 5,546 | 2,699 (48.7) | 2,847 (51.3) | 1.14 (1.05 – 1.23) |
| **> 100,000 €** | 97 | 44 (45.7) | 53 (54.6) | 1.00 (0.67 – 1.50) |
| **Area of residence** |  |  |  |  |
| **Rural** | 6,447 | 2,801 (47.7) | 3,070 (52.3) | 1 (ref.) |
| **Urban** | 4,364 | 2,269 (45.9) | 2,771 (54.1) | 0.93 (0.86-1.00) |

^a^ Adjusted simultaneously for all the variables included in the table

OR, Odds Ratio

**Supplementary Table 7. Odds Ratio  and 95% confidence interval ( OR 95%CI) of complete data at 12 years  according to baseline characteristics**

|  | **Total**  **n = 10,811**  **n (%)** | **Measured**  **n = 2,754 (25.5)**  **n (%)** | **Not measured**  **n = 8,057 (74.5)**  **n (%)** | **Adjusted OR^a^** |
| --- | --- | --- | --- | --- |
| **Sex** |  |  |  |  |
| **Boys** | 5,498 | 1,407 (25.6) | 4.091 (74.4) | 1 (ref.) |
| **Girls** | 5,313 | 1,347 (25.3) | 3,966 (74.7) | 0.99 (0.90 - 1.08) |
| **Weight status at 2-5 years of age** |  |  |  |  |
| **Underweight** | 78 | 13 (16.7) | 65 (83.3) | 0.61 (0.33 – 1.12) |
| **Normal weight** | 7,628 | 1,893 (24.8) | 5,735 (75.2) | 1 (ref.) |
| **At risk of overweight** | 2,291 | 608 (26.5) | 1,683 (73.5) | 1.10 (0.99 - 1.23) |
| **Overweight/Obese** | 814 | 240 (29.5) | 574 (70.5) | 1.27 (1.08– 1.49) |
| **Annual household income** |  |  |  |  |
| **Basic income** | 510 | 106 (20.8) | 404 (79.2) | 0.77 (0.61 – 0.96) |
| **Less than 18,000 €** | 4,657 | 1,220 (26.2) | 3,437 (73.8) | 1 (ref.) |
| **18.000€ - < 100.000 €** | 5,546 | 1,410 (25.4) | 4,136 (74.6) | 0.99 (0.90 – 1. 07) |
| **> 100,000 €** | 97 | 17 (17.5) | 80 (82.5) | 0.63 (0.37 – 1.07) |
| **Area of residence** |  |  |  |  |
| **Rural** | 6,447 | 1,705 (29.0) | 4,166 (71.0) | 1 (ref.) |
| **Urban** | 4,364 | 1,049 (21.2) | 3,891 (78.8) | 0.66 (0.60-0.72) |

^a^ Adjusted simultaneously for all the variables included in the table

OR, Odds Ratio

**Supplementary Table 8. Odds Ratio  and 95% confidence interval ( OR 95%CI) of complete data at 13 years according to baseline characteristics**

|  | **Total**  **n = 10,811**  **n (%)** | **Measured**  **n = 1,790 (16.6)**  **n (%)** | **Not measured**  **n = 9,021 (83.4)**  **n (%)** | **Adjusted OR^a^** |
| --- | --- | --- | --- | --- |
| **Sex** |  |  |  |  |
| **Boys** | 5,498 | 985 (17.9) | 4.513 (82.1) | 1 (ref.) |
| **Girls** | 5,313 | 805 (15.1) | 4,508 (84.9) | 0.82 (0.74 – 0.90) |
| **Weight status at 2-5 years of age** |  |  |  |  |
| **Underweight** | 78 | 11 (14.1) | 67 (85.9) | 0.81 (0.42 – 1.55) |
| **Normal weight** | 7,628 | 1,250 (16.4) | 6,378 (83.6) | 1 (ref.) |
| **At risk of overweight** | 2,291 | 364 (15.9) | 1,927 (84.1) | 0.95 (0.84 - 1.09) |
| **Overweight/Obese** | 814 | 165 (20.3) | 649 (79.7) | 1.29 (1.07– 1.55) |
| **Annual household income** |  |  |  |  |
| **Basic income** | 510 | 75 (14.7) | 435 (85.3) | 0.82 (0.63 – 1.07) |
| **Less than 18,000 €** | 4,657 | 764 (16.4) | 3,893 (83.6) | 1 (ref.) |
| **18.000€ - < 100.000 €** | 5,546 | 928 (16.7) | 4,618 (83.3) | 1.00 (0.91 – 1. 12) |
| **> 100,000 €** | 97 | 23 (23.7) | 74 (76.3) | 1.57 (0.97 – 2.53) |
| **Area of residence** |  |  |  |  |
| **Rural** | 6,447 | 819 (13.9) | 5,052 (86.1) | 1 (ref.) |
| **Urban** | 4,364 | 971 (19.7) | 3,979 (80.3) | 1.51 (1.36-0.67) |

^a^ Adjusted simultaneously for all the variables included in the table

OR, Odds Ratio

**Supplementary Table 9. Odds Ratio  and 95% confidence interval ( OR 95%CI) of complete data at 14 years according to baseline characteristics**

|  | **Total**  **n = 10,811**  **n (%)** | **Measured**  **n = 3,153 (29.2)**  **n (%)** | **Not measured**  **n = 7,658 (70.8)**  **n (%)** | **Adjusted OR^a^** |
| --- | --- | --- | --- | --- |
| **Sex** |  |  |  |  |
| **Boys** | 5,498 | 1,617 (29.4) | 3,881 (70.6) | 1 (ref.) |
| **Girls** | 5,313 | 1,536 (28.9) | 3,777 (71.1) | 0.97 (0.89 – 1.05) |
| **Weight status at 2-5 years of age** |  |  |  |  |
| **Underweight** | 78 | 18 (23.1) | 60 (76.9) | 0.73 (0.43 – 1.24) |
| **Normal weight** | 7,628 | 2,233 (29.3) | 5,395 (70.7) | 1 (ref.) |
| **At risk of overweight** | 2,291 | 684 (29.9) | 1,607 (70.1) | 1.03 (0.93 - 1.14) |
| **Overweight/Obese** | 814 | 218 (26.8) | 596 (73.2) | 0.90 (0.76– 1.05) |
| **Annual household income** |  |  |  |  |
| **Basic income** | 510 | 117 (22.9) | 393 (77.1) | 0.76 (0.61 – 0.95) |
| **Less than 18,000 €** | 4,657 | 1,296 (27.8) | 3,361 (72.2) | 1 (ref.) |
| **18.000€ - < 100.000 €** | 5,546 | 1,708 (30.8) | 3,838 (69.2) | 1.14 (1.05 – 1.24) |
| **> 100,000 €** | 97 | 32 (33.0) | 65 (67.0) | 1.26 (0.82 – 1.93) |
| **Area of residence** |  |  |  |  |
| **Rural** | 6,447 | 1,644 (28.0) | 4,227 (72.0) | 1 (ref.) |
| **Urban** | 4,364 | 1,509 (30.5) | 3,431 (69.5) | 1.13 (1.04-1.22) |

^a^ Adjusted simultaneously for all the variables included in the table

OR, Odds Ratio

**Supplementary Table 10. Complete data** **and IPW adjusted prevalence and 95% confidence interval for every age between 6 to 14 years.**

|  | | **Underweight** | **Normal weight** | **Overweight** | **Obese** |
| --- | --- | --- | --- | --- | --- |
| **6 years; n = 7,688 (71.1)** | |  |  |  |  |
|  | Complete data | 0.88 % (0.70–1.12) | 68.48% (67.44–69.51) | 19.99 % (19.11–20.90) | 10.64% (9.97–11.35) |
|  | IPW | 0.96 % (0.73–1.19) | 68.70% (67.65–69.73) | 19.68 % (18.80–20.57) | 10.66% (9.97–11.35) |
| **7 years; n = 2,456 (22.7)** | |  |  |  |  |
|  | Complete data | 1.06 % (0.72–1.55) | 62.13% (60.20–64.03) | 22.48 % (20.87–24.17) | 14.33% (13.00–15.78) |
|  | IPW | 1.07 % (0.66–1.49) | 63.35% (61.44–65.25) | 22.32% (20.68–23.97) | 13.25 % (11.95–14.56) |
| **8 years; n = 7,403 (68.5)** | |  |  |  |  |
|  | Complete data | 1.16 % (0.94–1.43) | 61.35% (60.24–62.46) | 24.00 % (23.04–24.99) | 13.48% (12.72–14.28) |
|  | IPW | 1.17 % (0.92–1.42) | 61.44% (60.33–62.55) | 23.89 % (22.92–24.86) | 13.50% (12.72–14.28) |
| **9 years; n = 2,046 (18.9)** | |  |  |  |  |
|  | Complete data | 1.76 % (1.27–2.43) | 55.91% (53.75–58.05) | 24.24 % (22.43–26.15) | 18.08% (16.47–19.81) |
|  | IPW | 1.90 % (1.26–2.54) | 58.62% (56.40–60.85) | 23.31 % (21.40–25.21) | 16.17 % (14.55–17.78) |
| **10 years; n = 4,476 (41.4)** | |  |  |  |  |
|  | Complete data | 1.43% (1.12–1.82) | 59.79% (58.34–61.22) | 25.49% (24.21–26.77) | 13.29% (12.33–14.32) |
|  | IPW | 1.55% (1.16–1.94) | 60.46% (59.02–61.90) | 25.35% (24.08–26.63) | 12.63% (11.67–13.59) |
| **11 years; n = 5070 (46.9)** | |  |  |  |  |
|  | Complete data | 2.43% (2.00–2.84) | 62.27% (60.93–63.60) | 24.77% (23.58–25.96) | 10.53% (9.72–11.41) |
|  | IPW | 2.42% (2.00–2.84) | 62.51 % (61.18–63.84) | 24.62% (23.43–25.80) | 10.45% (9.60–11.29) |
| **12 years; n = 2,753 (25.5)** | |  |  |  |  |
|  | Complete data | 2.72% (2.18–3.40) | 62.64% (60.81–64.43) | 23.60 % (22.05–25.23) | 11.04% (9.92–12.27) |
|  | IPW | 3.17% (2.43–3.91) | 63.49 % (61.64–65.34) | 22.94 % (21.34–24.54) | 10.39% (9.25–11.54) |
| **13 years; n = 1,790 (16.6)** | |  |  |  |  |
|  | Complete data | 2.73% (2.07–3.60) | 66.82% (64.60–68.96) | 21.23% (19.39–23.19) | 9.22% (7.96–10.65) |
|  | IPW | 2.75% (1.97–3.53) | 67.84% (65.63–70.04) | 20.89% (18.97–22.82) | 8.51% (7.23–9.79) |
| **14 years; n = 3,153 (29.2)** | |  |  |  |  |
|  | Complete data | 2.44% (1.96–3.04) | 73.39% (71.82–74.90) | 17.57% (16.28–18.93) | 6.60% (5.78–7.51) |
|  | IPW | 2.43% (1.89–2.97) | 72.98% (71.41–74,54) | 17.68% (16.34–19.02) | 6.89% (5.99–7.80) |
